# A novel nomogram for anastomotic leakage after surgery for rectal cancer: a retrospective study

**DOI:** 10.7717/peerj.14437

**Published:** 2022-11-28

**Authors:** Tingzhen Li, Jianglong Huang, Purun Lei, Xiaofeng Yang, Zehong Chen, Peng Chen, Jiancheng Zhai, Xuefeng Guo, Hongbo Wei

**Affiliations:** 1 Department of Gastrointestinal Surgery, The Third Affiliated Hospital, Sun Yat-sen University, Guangzhou, Guangdong, China; 2 Department of Endoscopic Surgery, The Sixth Affiliated Hospital, Sun Yat-sen University, Guangzhou, Guangdong, China

**Keywords:** Rectal cancer, Anastomotic leakage, Nomogram, Prediction model

## Abstract

**Background:**

Anastomotic leakage remains one of the most common serious complications after rectal cancer surgery. How to predict its occurrence and prevent it remains largely elusive.

**Objective:**

This study aimed to identify the risk factors of anastomotic leakage and construct a nomogram for predicting postoperative anastomotic leakage in patients with rectal cancer.

**Methods:**

The data of 406 patients with rectal cancer after gastrointestinal surgery in the Third Affiliated Hospital of Sun Yat-sen University from January 2011 to May 2020 were collected (243 in the training set and 163 in the testing set). Logistic regression was applied to determine the risk factors of postoperative anastomotic leakage of rectal cancer, and a nomogram prediction model was thus established. Predictive performance of the nomogram was evaluated by C-index and area under the receiver-operating characteristic (ROC) curve.

**Results:**

Logistic regression analysis showed that preoperative bowel obstruction (odds ratio [OR] = 12.846, 95% confidence interval CI [1.441–114.54], *p* = 0.022) and early first defecation after surgery (OR = 0.501, 95% CI [0.31–0.812], *p* = 0.005) were independent risk factors, which could be used to develop a nomogram to predict the occurrence of anastomotic leakage accurately. The evaluation of the prediction model shows that the C-index value of the model was 0.955, the area under the ROC curve (AUC) of the training set was 0.820, and the testing set was 0.747, whereas the optimal cut-off point based on the nomogram score was 174.6.

**Conclusion:**

This nomogram had a good prediction ability for postoperative anastomotic leakage in patients with rectal cancer. It can provide a reference for perioperative treatment and the selection of surgical methods to promote individualized and accurate treatment.

## Introduction

Colorectal cancer (CRC) is the second leading cause of cancer death worldwide (Siegel, Miller & Jemal, 2018). In China, CRC is the second most common cancer and the fourth leading cause of cancer death. It was estimated that there were 408,000 new cases of colorectal cancer and 195,600 deaths in 2016 (Zheng et al., 2022). According to previous reports, there are many treatment methods for CRC, but surgery remains the leading method to achieve a radical cure (Benson et al., 2020). Nevertheless, there are many complications, especially anastomotic leakage (AL), which is a common serious complication after anterior resection of rectal cancer, with an incidence of 2.4% ∼27.0% and a mortality rate of 18% (Matsubara et al., 2014; Matthiessen et al., 2007a; Matthiessen et al., 2007b; Platell et al., 2007).

AL may lead to reoperation, delayed discharge and chemotherapy time, increased incidence of complications and mortality, and risk of local recurrence (Ha, Kim & Lee, 2017; Krarup et al., 2014; Smith et al., 2012). Compared with open surgery, the incidence rate of AL in laparoscopic surgery has decreased, but there is no significant difference in AL incidence between surgical approaches (Sciuto et al., 2018). In recent years, anastomotic instruments and surgical techniques have developed greatly, but the incidence of AL has not decreased significantly (Shearer et al., 2013).

Therefore, it is necessary to develop a model that can predict the postoperative AL of patients with rectal cancer and identify risk reduction factors during treatment. The high-risk factors of AL have been widely discussed and studied. These factors include age, male, comorbidity, preoperative radiotherapy, emergency operation, and so on (Parthasarathy et al., 2017; Sparreboom et al., 2018; Zhou et al., 2019). Many researchers have tried to predict AL, which has a guiding role in risk assessment and decision-making during treatment (Eveno et al., 2016; Hoshino et al., 2018; Waterland et al., 2016). Previous large-scale case reports have often included single-center or different multicenter operation teams, with different inclusion and exclusion conditions, which objectively leads to bias in the research results. The advantage of our study is that we focused on the AL of patients with rectal cancer after surgery under the same surgical team. In addition, the model we created was evaluated in training and testing sets, which confirmed its good prediction ability. Therefore, we analyzed the clinical data of rectal cancer surgery performed by the same surgical team, discussed the influencing factors of AL after rectal cancer surgery, and established a nomogram to predict the occurrence of AL after rectal cancer surgery. Eventually, clinicians can use nomograms to treat rectal cancer patients with individualized treatment to provide a reference basis for the prevention and treatment of AL and take effective intervention measures in time during the perioperative period.

## Materials and Methods

### Patient selection

The retrospective study was approved by the ethics committee of the Third Affiliated Hospital of Sun Yat-sen University (approval No.: [2019]02-008-01). The data obtained does not contain a patient identifier. Patient data is retrieved from our hospital database without intervention, so there is no need to send patient information. This study is in line with the declaration of Helsinki. Inclusion criteria were complete medical records, pathological examination confirmed rectal cancer, open or laparoscopic anterior resection of rectal cancer, and total mesorectal excision (TME) followed for middle and lower rectal cancer. Exclusion criteria were abdominal metastases and patients who underwent abdominal perineal resection (miles) or Hartmann surgery. From the medical records of our institution, we collected data from the medical records of 406 consecutive patients after rectal cancer surgery at the Department of Gastrointestinal Surgery, The Third Affiliated Hospital, Sun Yat-Sen University from January 2011 to May 2020. Clinical data were collected and added to the database. The patients were divided into two groups: those with AL and those without. Enrolled patients were randomly divided into a training set and testing set at a ratio of 6:4.

Clinical data were collected from medical records. All surgery was performed by clinical colorectal surgeons. All patients underwent comprehensive preoperative evaluation, including physical examination, imaging examination, laboratory examination, and colonoscopy with biopsy. Perioperative management was performed according to the guidelines of the National Comprehensive Cancer Network. The American Joint Committee on Cancer staging systems (8th Edition) was used to determine the staging of tumors (Zhang et al., 2018).

### Definition of AL

In our study, AL was defined as the interruption and defect of intestinal wall integrity at the site of the colon rectum or colon anal anastomosis, making the internal and external compartments connected. According to the grade of anastomotic leakage of the International Study Group of Rectal Cancer in 2010 (Rahbari et al., 2010), AL can be divided into three grades: A, which only shows imaging AL without special treatment; B, AL with clinical manifestations of abdominal pain, fever, purulent or fecal residue like drainage from the anus, drainage tube or vagina needs conservative treatment, such as antibiotics or drainage; and C, AL with clinical manifestations such as peritonitis and sepsis needing secondary surgical treatment. AL in this study was diagnosed according to the grade C standard.

### Selection of clinical factors and establishment of a clinical model

Clinical factors of AL and No AL groups included pT stage, pN stage, pM stage, pTNM stage, gender, age, BMI, alcohol intake, smoking, diabetes, CEA, CA199, neoadjuvant chemotherapy, neoadjuvant radiotherapy, preoperative albumin, preoperative Hb, bowel obstruction, distance from the anal verge, tumor diameter, ASA score, oral antibiotic, surgical approach, emergency operation, operating time, LCA preservation, intraoperative blood loss, intraoperative lavage, number of linear stapler firing, prophylactic ileostomy, trans-anal drainage, defecation, and drainage tube removal time (Table 1). Because it was more feasible to use two groups for some factors in clinical practice and for statistical analyses, we converted some continuous variables into classified variables, including age, CEA, CA199, distance from anal margin, tumor diameter, operation time, and the cut-off values of these factors (Table 1). After univariate analysis, factors with *P* < 0.1 and greater significance in clinical practice in the training set were introduced into the stepwise logistic regression, and the Akaike information criterion (AIC) was used as the stopping rule to construct the clinical model (Collins et al., 2015).

**Table 1:** Univariate analysis of factors for AL in the training and testing sets.

Clinical factors	Training set (*n* = 243)			Testing set (*n* = 163)		
	No AL (*n* = 228)	AL (*n* = 15)	*p* value	No AL (*n* = 157)	AL (*n* = 6)	*p* value
pT stage			0.079			0.506
T0	7(3.07%)	1(6.67%)		3(1.91%)	0(0.00%)	
T1	20(8.77%)	4(26.67%)		13(8.28%)	0(0.00%)	
T2	41(17.98%)	1(6.67%)		31(19.75%)	0(0.00%)	
T3	51(22.37%)	1(6.67%)		46(29.30%)	1(16.67%)	
T4	109(47.81%)	8(53.33%)		64(40.76%)	5(83.33%)	
pN stage			0.062			0.690
N0	142(62.28%)	5(33.33%)		97(61.78%)	4(66.67%)	
N1	54(23.68%)	7(46.67%)		43(27.39%)	1(16.67%)	
N2	32(14.04%)	3(20.00%)		17(10.83%)	1(16.67%)	
pM stage			0.440			0.398
M0	205(89.91%)	12(80.00%)		145(92.36%)	5(83.33%)	
M1	23(10.09%)	3(20.00%)		12(7.64%)	1(16.67%)	
pTNM stage			0.059			1.000
<III	136(59.65%)	5(33.33%)		93(59.24%)	4(66.67%)	
≥III	92(40.35%)	10(66.67%)		64(40.76%)	2(33.33%)	
Gender			0.268			0.503
Male	144(63.16%)	12(80.00%)		96(61.15%)	5(83.33%)	
Female	84(36.84%)	3(20.00%)		61(38.85%)	1(16.67%)	
Age, years			0.791			0.120
<60	142(62.28%)	10(66.67%)		94(59.87%)	6(100.00%)	
≥60	86(37.72%)	5(33.33%)		63(40.13%)	0(0.00%)	
BMI, kg/m^2^			0.873			0.592
<25	194(85.09%)	12(80.00%)		132(84.08%)	6(100.00%)	
≥25	34(14.91%)	3(20.00%)		25(15.92%)	0(0.00%)	
Alcohol intake			0.325			0.264
Yes	20(8.77%)	3(20.00%)		7(4.46%)	1(16.67%)	
No	208(91.23%)	12(80.00%)		150(95.54%)	5(83.33%)	
Smoking			0.286			0.638
Yes	55(24.12%)	6(40.00%)		27(17.20%)	2(33.33%)	
No	173(75.88%)	9(60.00%)		130(82.80%)	4(66.67%)	
Diabetes			0.970			0.530
Yes	24(10.53%)	1(6.67%)		18(11.46%)	1(16.67%)	
No	204(89.47%)	14(93.33%)		139(88.54%)	5(83.33%)	
CEA, µg/L			0.433			0.833
<5	152(66.67%)	12(80.00%)		111(70.70%)	5(83.33%)	
≥5	76(33.33%)	3(20.00%)		46(29.30%)	1(16.67%)	
CA199,U/L			0.946			1.000
<35	192(84.21%)	12(80.00%)		136(86.62%)	6(100.00%)	
≥35	36(15.79%)	3(20.00%)		21(13.38%)	0(0.00%)	
Neoadjuvant chemotherapy			1.000			0.433
Yes	69(30.26%)	5(33.33%)		42(26.75%)	3(50.00%)	
No	159(69.74%)	10(66.67%)		115(73.25%)	3(50.00%)	
Neoadjuvant radiotherapy						1.000
Yes	0(0.00%)	0(0.00%)		3(1.91%)	0(0.00%)	
No	228(100.00%)	15(100.00%)		154(98.09%)	6(100.00%)	
Preoperative albumin, g/L			1.000			1.000
>30	226(99.12%)	15(100.0%)		151(96.18%)	6(100.0%)	
≤30	2(0.88%)	0(0.00%)		6(3.82%)	0(0.00%)	
Preoperative Hb, g/L			0.799			0.877
≤110	61(26.75%)	5(33.33%)		60(38.22%)	3(50.00%)	
>110	167(73.25%)	10(66.67%)		97(61.78%)	3(50.00%)	
Bowel obstruction			0.142			0.045
Yes	9(3.95%)	2(13.33%)		8(5.10%)	2(33.33%)	
No	219(96.05%)	13(86.67%)		149(94.90%)	4(66.67%)	
Distance from anal verge, cm			0.269			1.000
≤7	82(39.74%)	8(53.33%)		71(45.22%)	3(50.00%)	
>7	146(64.04%)	7(46.67%)		86(54.78%)	3(50.00%)	
Tumor diameter, cm			0.710			0.830
<5	170(74.56%)	10(66.67%)		124(78.98%)	4(66.67%)	
≥5	58(25.44%)	5(33.33%)		33(21.02%)	2(33.33%)	
ASA score			1.000			1.000
<3	216(94.74%)	15(100.00%)		152(96.82%)	6(100.00%)	
≥3	12(5.26%)	0(0.00%)		5(3.18%)	0(0.00%)	
Oral antibiotic			0.473			1.000
Yes	74(3.46%)	3(20.00%)		49(31.21%)	2(33.33%)	
No	154(67.54%)	12(80.00%)		108(68.79%)	4(66.67%)	
Surgical approach			1.000			0.373
Open	13(5.70%)	0(0.00%)		11(7.01%)	1(16.67%)	
Laparoscopy	215(94.30%)	15(00.00%)		146(92.99%)	5(83.33%)	
Emergency operation			1.000			0.173
Yes	7(3.07%)	0(0.00%)		4(2.55%)	1(16.67%)	
No	221(96.93%)	15(00.00%)		153(97.45%)	5(83.33%)	
Operating time, min			0.424			0.484
<180	87(38.16%)	4(26.67%)		62(39.49%)	1(16.67%)	
≥180	141(61.84%)	11(73.33%)		95(60.51%)	5(83.33%)	
LCA preserving			1.000			1.000
Yes	8(3.51%)	0(0.00%)		2(1.27%)	0(0.00%)	
No	220(96.49%)	15(100.00%)		155(98.73%)	6(100.00%)	
Intraoperative blood loss,mL			0.970			0.132
<100	204(89.47%)	14(93.33%)		141(89.81%)	4(66.67%)	
≥100	24(10.53%)	1(6.67%)		16(10.19%)	2(33.33%)	
Intraoperative lavage			1.000			1.000
Yes	2(0.88%)	0(0.00%)		3(1.91%)	0(0.00%)	
No	226(99.12%)	15(100.00%)		154(98.09%)	21(100.00%)	
Number of linear stapler firing			0.294			0.026
0	2(0.88%)	0(0.00%)		0(0.00%)	0(0.00%)	
1	162(71.05%)	8(53.33%)		105(66.88%)	2(33.33%)	
2	63(27.63%)	7(46.67%)		51(32.48%)	3(50.00%)	
3	1(0.44%)	0(0.00%)		1(0.64%)	1(16.67%)	
Number of linear stapler firing			0.215			0.208
≥2	64(28.07%)	7(46.67%)		52(33.12%)	4(52.4%)	
<2	164(71.93%)	8(53.33%)		105(66.88%)	2(66.67%)	
Prophylactic ileostomy			1.000			1.000
Yes	28(12.28%)	2(13.33%)		21(13.38%)	0(0.00%)	
No	200(87.72%)	13(86.67%)		136(86.62%)	6(100.00%)	
Trans-anal drainage			1.000			1.000
Yes	78(34.21%)	5(33.33%)		68(43.31%)	3(50.00%)	
No	150(65.79%)	10(66.67%)		89(56.69%)	3(50.00%)	
Defecation	3.298 ± 1.573	2.267 ± 1.624	0.030	3.567 ± 1.520	1.500 ± 0.837	0.001
Drainage tube removal time	3.557 ± 1.899	3.267 ± 2.154	0.570	3.427 ± 1.634	3.500 ± 2.345	0.916

**Notes.**

Abbreviations AL Anastomotic leakage BMI body mass index ASA American Society of Anesthesiologists HGB hemoglobin LCA left colic artery

### Construction of a clinical nomogram

The selected clinical factors were introduced into logistic regression analysis to establish a clinical model. According to the results of logistic regression analysis in the training set, a clinical nomogram was constructed as a visual and quantitative method to predict the individual risk of AL.

### Performance evaluation of established models

The nomogram of the model was constructed by using the factors derived from the training set, and the prediction ability of the model was evaluated in the training set and testing set. In the training and testing sets, the diagnostic performance of the clinical model and clinical nomogram were evaluated according to the C-index and the area under the receiver operator characteristic (ROC) curve (AUC).

### Statistical analysis

R programming language (version 3.4.2) and SPSS 25.0 software (IBM) were used for the data analysis. The data are expressed as the mean ± standard deviation. In this study, the chi-square test, Fisher exact test, and *t*-test were used to compare the data between groups. After screening by univariate analysis, factors with *p* < 0.1 in the training set and greater significance in clinical practice were introduced into the stepwise logistic regression.

The ROC curve of the model was drawn using the “pROC” package. The clinical nomogram was constructed by the “rms” package. The predictive performance of the model was assessed by the AUC. The internal verification of the nomogram used 1,000 bootstrap resampling to reflect the performance of the model. In addition, the relationship between observation frequency and prediction probability was described by a calibration curve. The best cut-off point of the nomogram score was reflected by the Youden index. When *P* < 0.05, the difference was considered statistically significant.

## Results

### Clinical factors and the established clinical model

**Figure 1:**
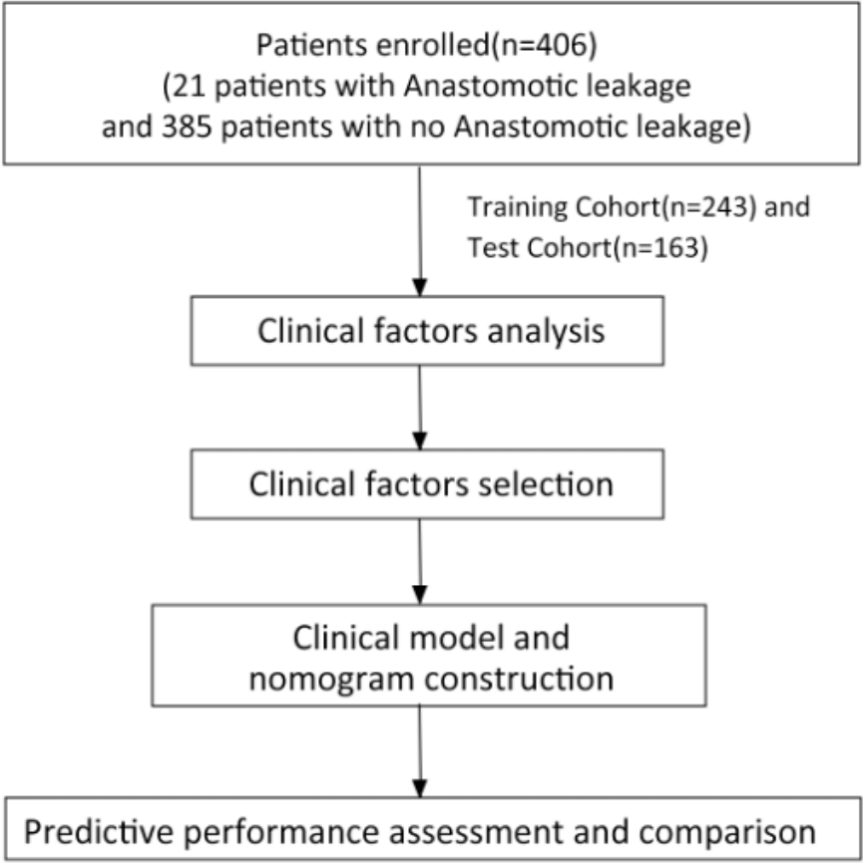
The study workflow.

A total of 406 patients who underwent rectal cancer surgery met the inclusion criteria and were enrolled in this study as shown in Fig. 1. Among them, 21 cases (5.17%) developed AL, all of which were grade C AL, including 17 males and four females. Most patients were male (257, 63.30%). Compared with patients without AL, those with AL had more preoperative bowel obstructions (19.05%), higher number of linear stapler firings (≥2), and shorter time to the first defecation after surgery (2.048 ±1.465). Clinical factors of the AL group and No AL group in the training and testing sets are shown in Table 1.

After univariate analysis, factors with *P* < 0.1 and greater significance in clinical practice in the training set were selected by stepwise logistic regression analysis. We used the AIC as a criterion in the stepwise logistic regression analysis and the model with the smallest AIC value was chosen. The AIC value of the selected model was 106.87 as shown in Table 2.

**Table 2:** The influencing factors for AL from logistic regression.

Variables	*β*	SE	OR[95% CI]	*p* value
Intercept	−0.391	1.292	–	0.762
pT stage (ref: 0 stage)				
1 stage	0.861	1.264	2.366[0.199–28.173]	0.495
2 stage	−1.628	1.517	0.196[0.01–3.838]	0.283
3 stage	−1.874	1.534	0.153[0.008–3.101]	0.222
4 stage	−0.552	1.199	0.576[0.055–6.032]	0.645
Gender (ref: male)	−1.157	0.747	0.314[0.073–1.359]	0.121
Defecation	−0.690	0.246	0.501[0.31–0.812]	0.005
Bowel obstruction	2.553	1.116	12.846[1.441–114.54]	0.022
Number of linear stapler firing	0.874	0.608	2.396[0.727–7.896]	0.151
Emergency operation	−16.741	1293.797	0.000[0.000–InF]	0.990

**Notes.**

AIC, 106.87.

### The constructed clinical nomogram

**Figure 2:**
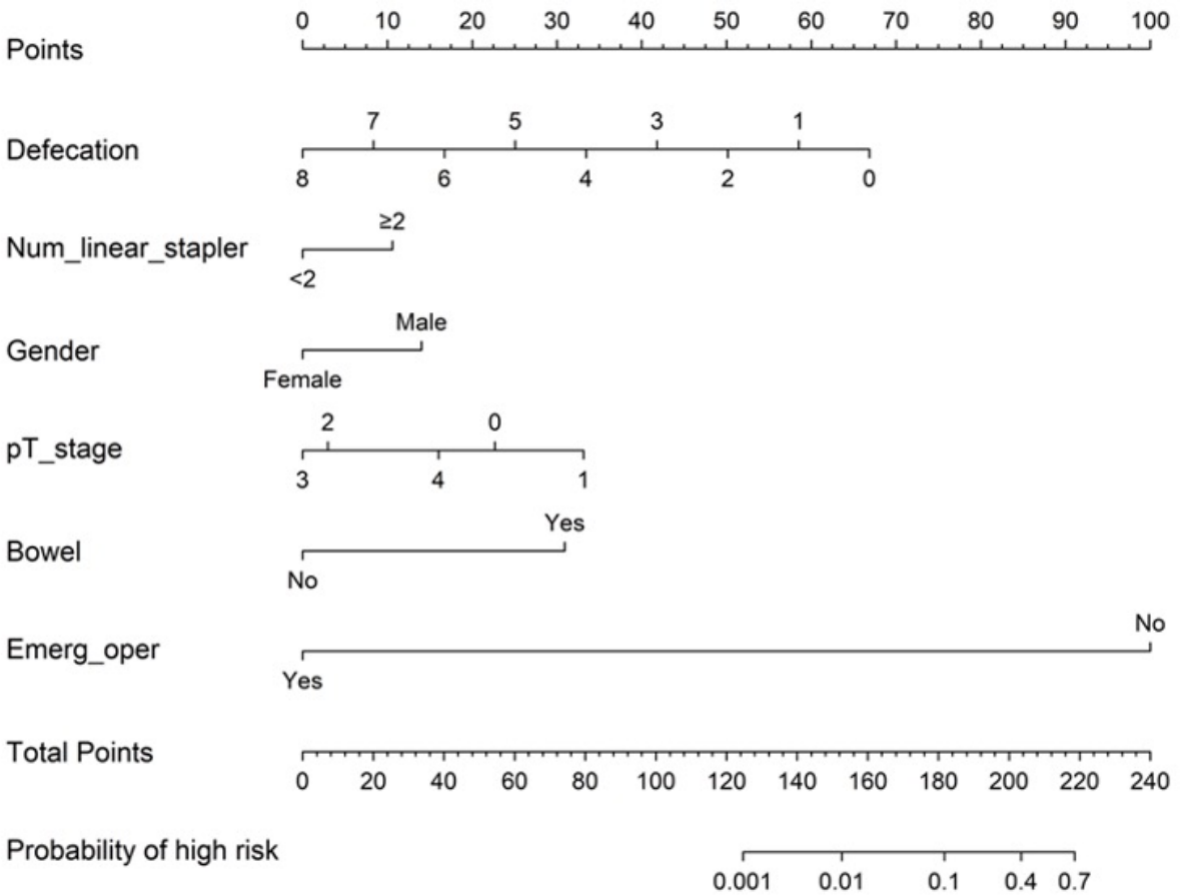
Nomogram for predicting postoperative AL (C-index 0.955). Defecation=Time to the first defecation after surgery; Num_linear_stapler= Number of linear stapler firing; Bowel= Preoperative bowel obstruction; Emerg_oper= Emergency operation. The incidence of AL was estimated by adding the factor scores.

After performing logistic regression analysis, a clinical nomogram was constructed to predict the risk of AL after rectal cancer surgery on the basis of pT stage, gender (odds ratio [OR] = 0.314, 95% confidence interval CI [0.073−1.359], *p* = 0.121), defecation (OR = 0.501, 95% CI [0.31−0.812], *p* = 0.005), bowel obstruction (OR = 12.846, 95% CI [1.441–114.54], *p* = 0.022), number of linear stapler firings (OR = 2.396, 95% CI [0.727−7.896], *p* = 0.151), and emergency operation (OR = 0.000, 95% CI [0.000–InF], *p* = 0.990). The nomogram is shown in Fig. 2. We calculated the sum of all variables on the total-point axis and then made a vertical line between the total-point axis and the resulting axis to estimate the estimated probability of AL. The nomogram score (Nomo score) of each patient was calculated as follows: Nomo score:

(1) Time to the first defecation after surgery: −8.35968 × defecation + 66.87744;

(2) Number of linear stapler firings (≥2):10.585;

(3) Gender(male):14.020;

(4) pT stage:0 stage:22.704, 1 stage:33.138, 2 stage:2.981, 3 stage:0.000, and 4 stage:16.015;

(5) Preoperative bowel obstruction (yes):30.924;

(6) Emergency operation (no): 100;

Probability of high risk = 1/(1+exp(-linear.predictors))

### Assessment of the performance of the established model

**Figure 3:**
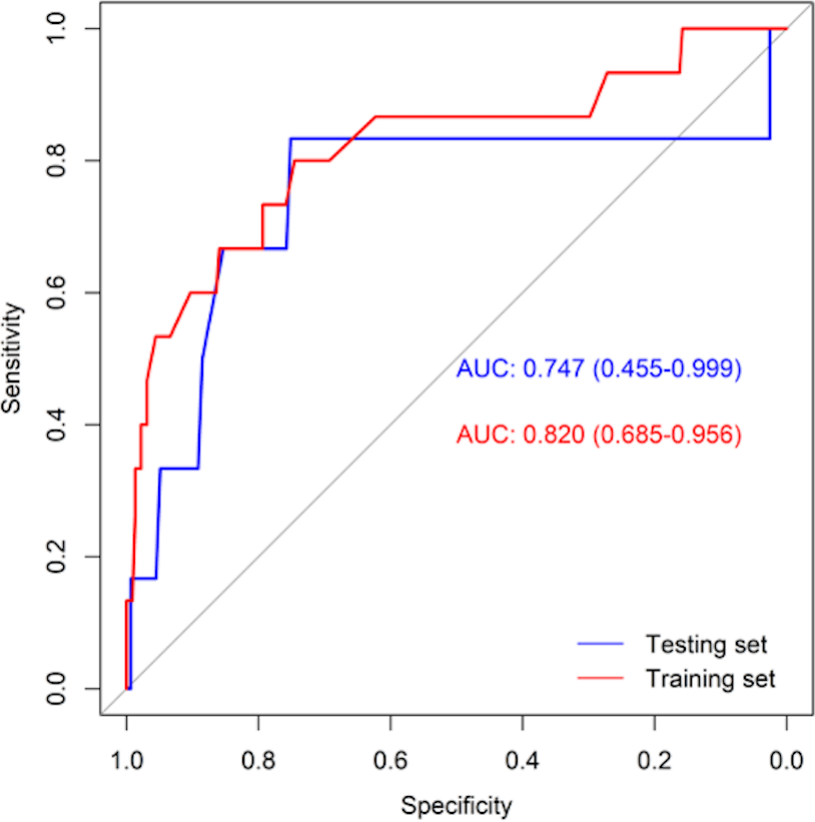
ROC curves of the clinical model in training set (red line) and testing set (blue line).

**Figure 4:**
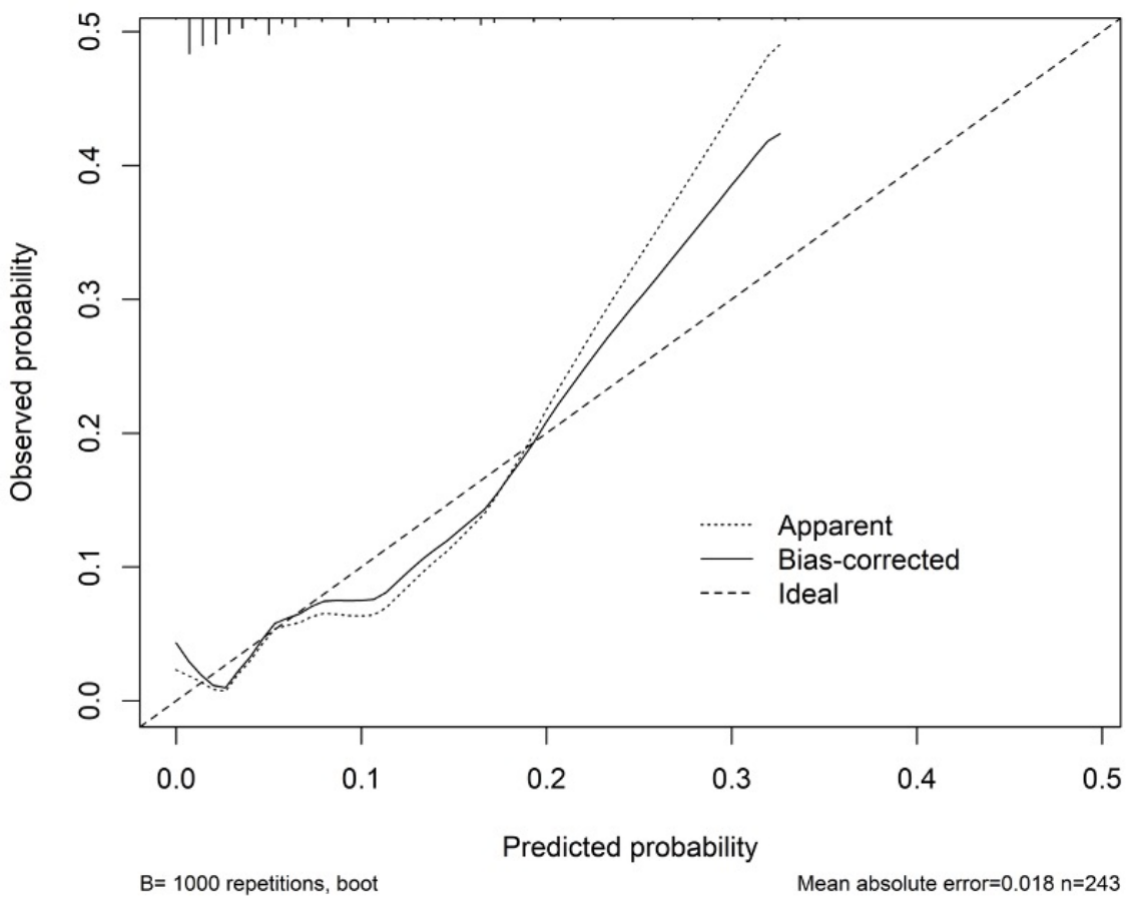
Calibration curve for the nomogram. The *x*-axis represents the predicted probability of AL. The *y*-axis represents the actual probability of AL. The ideal line represents a perfect prediction model. The apparent line symbolizes the performance of nomogram. The closer to the ideal line, the better the prediction performance.

**Figure 5:**
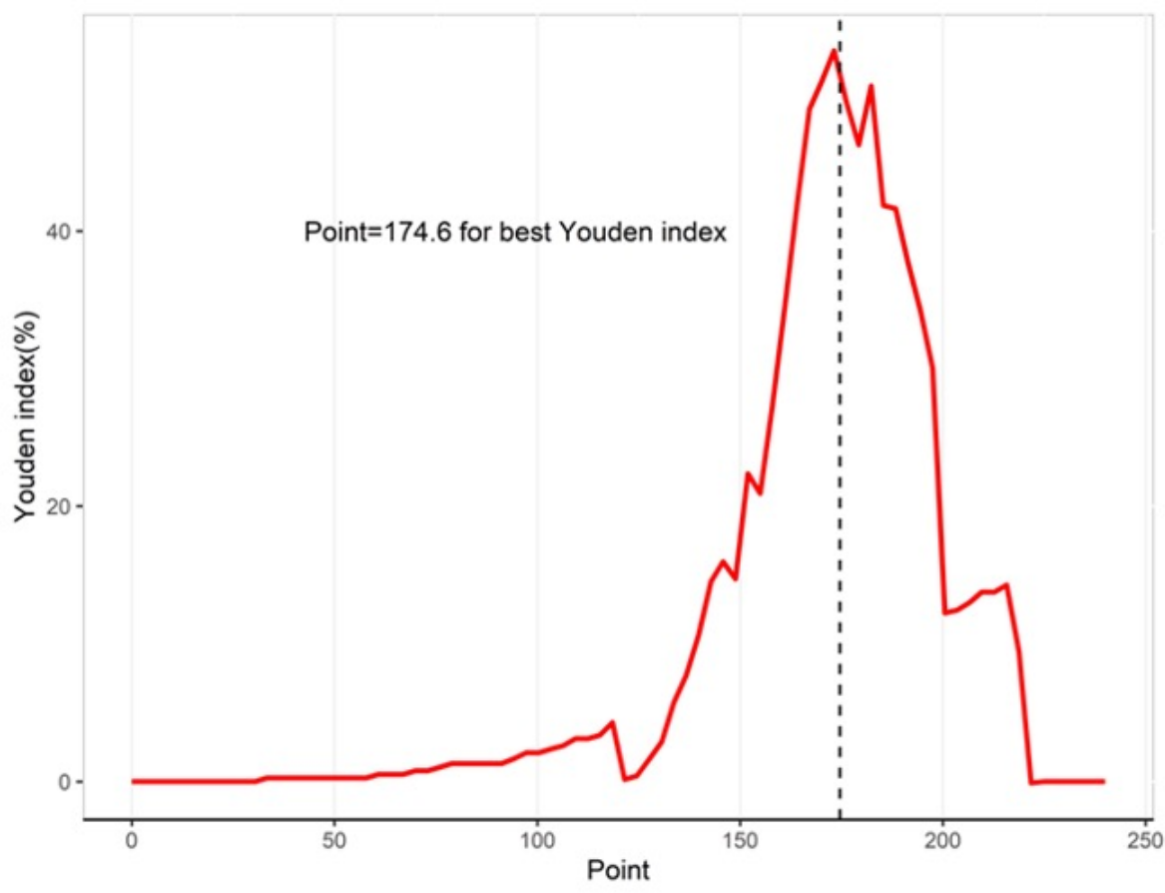
Youden indices for different cutoff point for the nomogram.

Table 3 shows the performance of the established clinical model. In the training set, the sensitivity was 80.0%, the specificity was 74.6%, and the accuracy was 74.9%. In the testing set, the sensitivity was 83.3%, the specificity was 75.2%, and the accuracy was 75.5%. The ROC curve of the clinical model is shown in Fig. 3. The ROC analysis results showed that the model had good prediction ability. The AUC value was 0.820 (95% CI [0.685–0.956]) in the training set, 0.747 (95% CI [0.455–0.999]) in the testing set, and 0.802 (95% CI [0.683–0.921]) in the full cohort. The C-index value of this model was 0.955, which shows it could predict the medium degree of the risk of AL. The calibration plot showed that the model was close to the ideal state, indicating that the calibration was good (Fig. 4). The best cut-off point based on the nomogram score was 174.6 (Fig. 5). The use of clinical nomograms to predict AL was proven to be beneficial.

**Table 3:** Assessment indices for the logistic regression.

Data set	AUC (95% CI)	Sensitivity	Specificity	Accuracy
Training set	0.820(0.685–0.956)	0.800	0.746	0.749
Testing set	0.747(0.455–0.999)	0.833	0.752	0.755
Full set	0.802(0.683–0.921)	0.810	0.748	0.751

## Discussion

In this study, we took several steps to evaluate the risk factors of AL after rectal cancer surgery and created a nomogram based on clinical factors as a method to predict AL after rectal cancer surgery. We retrospectively analyzed the clinical data of 406 patients with rectal cancer, divided them into a training set and testing set, and then performed an analysis on the training set and testing set. This study found that preoperative bowel obstruction and early first defecation after surgery were independent risk factors for AL. According to the above results, we established a prediction mathematical model. The C-index value of the model was 0.955, which showed its moderate prediction ability for AL risk. The AUC value of the prediction model was 0.820 in the training set and 0.747 in the testing set, which confirmed its good advantage in predicting patient risk. These findings may have clinical significance for carefully selecting patients for ileotomy according to our nomogram. Previous reports have described many potential risk factors for AL after low anterior resection of rectal cancer. Yao et al. (2014) created a nomogram to predict the risk of AL after laparoscopic anterior resection, including preservation of the left colonic artery, operation time, and tumor location. The prospective study of Hoshino et al. (2018) reported the independent risk factors of AL were preoperative serum albumin, gender, simultaneous resection of other organs, and tumor diameter and location, and established a nomogram to predict the risk of AL after anterior resection. Low anterior rectal resection was the focus of that study. In contrast, our study focused on the clinical data of rectal cancer surgery performed by the same surgical team and evaluated the model we created in the training set and the testing set. It provides a reference basis for clinicians for the prevention and treatment of AL and the selection of surgical schemes.

The results showed that the incidence of AL was 5.17%, and all were grade C leakage. Compared with the previous research results, the incidence of postoperative anastomotic leakage in this study was low. There are three possible reasons for this: (1) grade A and grade B AL were not included in this study. (2) This study only counted anastomotic leakage during hospitalization and omitted some patients with early and late anastomotic leakage. (3) This study was a single-center retrospective study with the same surgical team. The pathogenesis of AL remains unclear, and it is affected by many factors. Previous studies have found that many factors such as age, BMI, gender, tumor stage, tumor size, ASA score, preoperative radiotherapy and chemotherapy, operation time, malnutrition, and diabetes are related to AL (Parthasarathy et al., 2017). We found that in this study, preoperative bowel obstruction (*P* = 0.005) and early first defecation after surgery (*P* = 0.022) were independent risk factors for AL. The risk of AL with preoperative bowel obstruction was much higher than that without preoperative bowel obstruction. Patients with rectal cancer complicated with bowel obstruction are prone to postoperative complications due to insufficient food, impaired intestinal function, brittle tissue, edema and expansion of the intestinal wall, and even being water-electrolyte disorder and bacterial ectopic, which affects postoperative anastomotic healing, and significantly increases the incidence of AL (Ruggiero et al., 2011).

Some studies suggested that early first defecation after surgery is a high-risk factor for AL, which is similar to our results (Makela, Kiviniemi & Laitinen, 2003). The significant correlation between AL and early first defecation after surgery may be related to the increase of intraluminal pressure around the anastomosis. We speculate that the early first defecation after surgery will increase the pressure in the anastomotic cavity, increase the risk of anastomotic dehiscence, and allow excreta to flow out of the anastomotic site, thus increasing the occurrence of AL. It was reported that tumor stage is an important risk factor in the postoperative AL of rectal cancer (Sciuto et al., 2018). In our study, pathological T stage was not an independent risk factor. Patient-related factors, such as gender (Park et al., 2016) and age (Jung et al., 2008; Kumar et al., 2011), also had a great impact on AL. However, our study did not find significant differences in gender and age. Linear stapler firings ≥ 2 was also reported as an important risk factor for AL after rectal cancer surgery (Kim et al., 2009; Park et al., 2013). In the univariate analysis of our full data set, a number of linear stapler firings ≥ 2 was closely related to the occurrence of AL, but it was not an independent risk factor. This might be because the sample size was too small, and greater numbers are needed for future research.

Based on the results of logistic regression analysis, we drew a nomogram, including pT stage, gender, defecation, bowel obstruction, number of linear stapler firings, and emergency operation. Surgeons can use our nomogram to accurately estimate the risk of AL after rectal cancer surgery, because the nomogram is composed of clinical factors before and after surgery. It can facilitate surgeons to make better surgical plans. Diverting stoma or Hartmann’s procedure would be a good treatment for patients with AL risk according to the nomogram before surgery, such as patients with bowel obstruction. Prophylactic use of antidiarrheal drugs to delay time to the first defecation after surgery or placement of the anal canal is helpful to prevent AL. In addition, we should strengthen the monitoring of such patients and perioperative interventional therapy. In addition, we should pay close attention to the changes in their condition and take effective intervention measures in time during the perioperative period.

The advantage of our study was that it focused on the AL of patients with rectal cancer after surgery using the same surgical team. Second, the model we created was evaluated in the training set and testing set, which confirmed its good prediction ability. This study investigated a variety of factors related to AL and constructed a nomogram to better predict the risk of AL. However, the current research had some limitations. First, the postoperative AL we calculated was grade C leakage, which will lead to a low rate of postoperative AL. Second, due to the inherent defects of retrospective studies and the limited sample size (*n* = 406 patients), the limited sample may affect the veracity accuracy of the model. Therefore, we expect to conduct further prospective, multicenter randomized controlled studies to improve the practicability and reliability of our prediction model.

## Conclusions

In this study, we evaluated the risk factors of AL after rectal cancer surgery through logistic analysis and constructed a corresponding nomogram. We verified the nomogram and found that it had good prediction ability. The nomogram can accurately identify target patients and provide a reference for the selection of perioperative treatment and surgical methods to promote individualized and accurate treatment.

##  Supplemental Information

10.7717/peerj.14437/supp-1 Supplemental Information 1 R code

10.7717/peerj.14437/supp-2 Supplemental Information 2 Data The data of 406 patients with rectal cancer after gastrointestinal surgery in the Third Affiliated Hospital of Sun Yat-sen University from January 2011 to May 2020 were collected (243 in the training set and 163 in the testing set).

10.7717/peerj.14437/supp-3 Supplemental Information 3 A codebook to convert numbers to their respective factors for categorical data

10.7717/peerj.14437/supp-4 Supplemental Information 4 Table 4 Positive predictive value (PPV) and negative predictive value (NPV) for the clinical model

10.7717/peerj.14437/supp-5 Supplemental Information 5 Decision curve for training set (red line), testing set (yellow line), and full set (blue line)

## Additional Information and Declarations

### Data Availability

The following information was supplied regarding data availability: The raw measurements are available in the Supplementary Files.
