# Supplementary material for: A novel nomogram for anastomotic leakage after surgery for rectal cancer: a retrospective study
Source: PeerJ. 2022 Nov 28;10:e14437. doi: 10.7717/peerj.14437 (PMC9744139; doi:10.7717/peerj.14437)
Supplement: Supplemental Information 4 [file peerj-10-14437-s004.docx]

Table S1 **Positive predictive value (PPV) and negative predictive value (NPV) for the clinical model**

| Data set | PPV(95%CI) | NPV (95%CI) |
| --- | --- | --- |
| Training set | 0.222 (0.128, 0.667) | 0.983 (0.968, 0.995) |
| Testing set | 0.135 (0.078, 0.333) | 0.992 (0.976, 0.999) |
| Full set | 0.162 (0.114, 0.271) | 0.986 (0.975, 0.997) |
