# Supplementary figures and images for: A novel nomogram for anastomotic leakage after surgery for rectal cancer: a retrospective study
Source: PeerJ. 2022 Nov 28;10:e14437. doi: 10.7717/peerj.14437 (PMC9744139; doi:10.7717/peerj.14437)

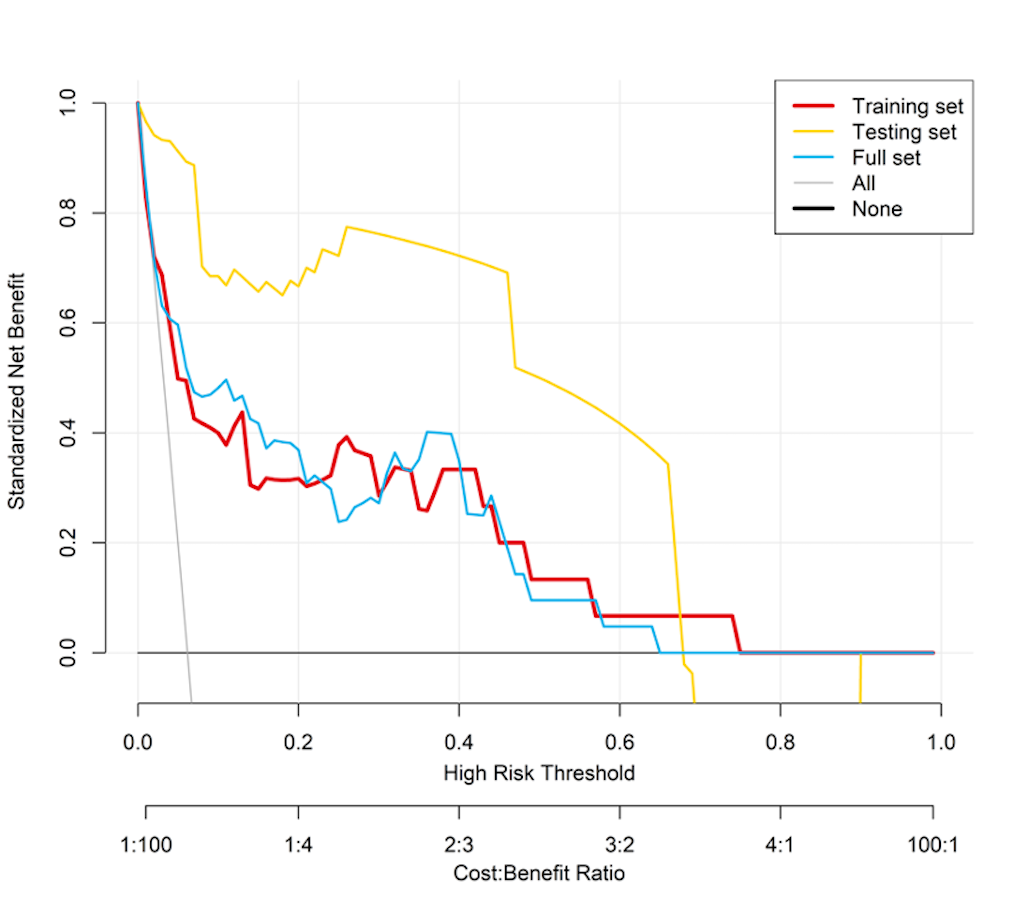

Supplement: Supplemental Information 5 [file peerj-10-14437-s005.png]
